# Short‐term, low‐level nitrogen deposition dampens a trophic cascade between bears and plants

**DOI:** 10.1002/ece3.4593

**Published:** 2018-10-25

**Authors:** Joshua B. Grinath

**Affiliations:** ^1^ Department of Biology Middle Tennessee State University Murfreesboro Tennessee; ^2^ Rocky Mountain Biological Laboratory Crested Butte Colorado; ^3^ Department of Biological Science Florida State University Tallahassee Florida

**Keywords:** ants, biogeochemistry, carnivores, commensalism, community ecology, food webs, herbivory, mutualism, nitrogen pollution, predation, species interactions, trophic structure

## Abstract

Human activities have substantially increased atmospheric nitrogen (N) deposition in ecosystems worldwide, often leading to higher plant quality for herbivores and greater herbivory. Predators frequently suppress herbivores and indirectly benefit plants via “trophic cascades”, and the strength of these interactions can also depend on N availability. However, the evidence for N deposition effects on cascades primarily comes from studies of high‐level N deposition. Most terrestrial ecosystems currently receive elevated, but low‐level N deposition, and it is unclear whether this subtle N enrichment has any effect on cascades. Here, I asked whether low‐level N deposition alters a trophic cascade from black bears to plants in Colorado. In this ecological network, bears indirectly benefit plants by consuming ants and suppressing positive effects of ants on herbivores. Using a three year N enrichment experiment, I assessed changes in this cascade by measuring plant and arthropod responses to simulated N deposition, bear damage to ant nests, and the presence of mutualist herbivores and ants. I found that low‐level N enrichment and bears had interacting effects on plant reproduction. In ambient N conditions, bears indirectly increased plant reproduction by causing ant nests to become inactive and suppressing positive ant effects on herbivores that were detrimental for plants. Yet, bear‐induced ant nest inactivity had no effect on plant reproduction in N‐enriched conditions. When N was added, ants had greater positive effects on herbivores, but herbivores had weak effects on plants, potentially because plants were more resistant to herbivores. Ultimately, the results indicate that N enrichment strengthened resource control of the community and weakened plant–herbivore interactions and the cascade from bears to plants. This study suggests that common rates of low‐level N deposition are changing the strength of trophic cascades and may have already altered resource versus consumer control of ecological community structure in many ecosystems.

## 1 INTRODUCTION

Before the twentieth century, most of the world experienced rates of atmospheric nitrogen (N) deposition that were near zero, but N deposition has become a major driver of global change as it has increased in many environments that were once limited by N availability (Dentener et al., 2006; Duce et al., 2008; Galloway et al., 2004, 2008 ; Vitousek et al., 1997). Nitrogen deposition can dramatically affect recipient ecosystems by altering plant performance and diversity (Bobbink et al., 2010; Clark & Tilman, 2008) and indirectly affecting species interactions at higher trophic levels (Meunier, Gundale, Sanchez, & Liess, 2016). By enhancing plant quality for herbivores, N deposition frequently intensifies herbivory (Throop & Lerdau, 2004); and with greater prey quality and availability resulting from N deposition, predators often respond by increasing in abundance and changing predatory behaviors (de Sassi, Staniczenko, & Tylianakis, 2012; Hagvar & Klanderud, 2009).

Much less is known about how N deposition influences the effects of higher trophic levels on plants. Trophic cascades, whereby predators suppress herbivores and indirectly benefit plants (Hairston, Smith, & Slobodkin, 1960), occur in ecosystems worldwide (Estes et al., 2011; Ripple et al., 2014) and a handful of studies have shown that N deposition can alter the magnitude of trophic cascades (Crowther et al., 2015; Hines, Reyes, & Gessner, 2016; Kardol, Spitzer, Gundale, Nilsson, & Wardle, 2016; Schmitz, 1994; Strauss, 1987). These studies have focused on assessing the impacts of very high rates of N deposition (>10 kg ha^−1^ year^−1^), which primarily occur in concentrated areas downwind of industrial and agricultural operations (Fenn et al, 2003; Galloway et al., 2008; Greaver et al., 2012). High N inputs often have transformative effects that reverberate throughout ecosystems (Galloway & Cowling, 2002), which can exhibit non‐linear and site‐specific response to increases in N subsidies (Knorr, Frey, & Curtis, 2005; Vivanco, Irvine, & Martiny, 2015). While high rates of N deposition occur regionally across the planet, elevated, but low‐level N deposition (5–10 kg ha^−1^ year^−1^) occurs at larger continental scales (Dentener et al., 2006; Galloway et al., 2004) and may have more widespread effects on ecosystems. Whether these relatively subtle rates of N enrichment can alter trophic cascades is uncertain.

**Figure 1:**
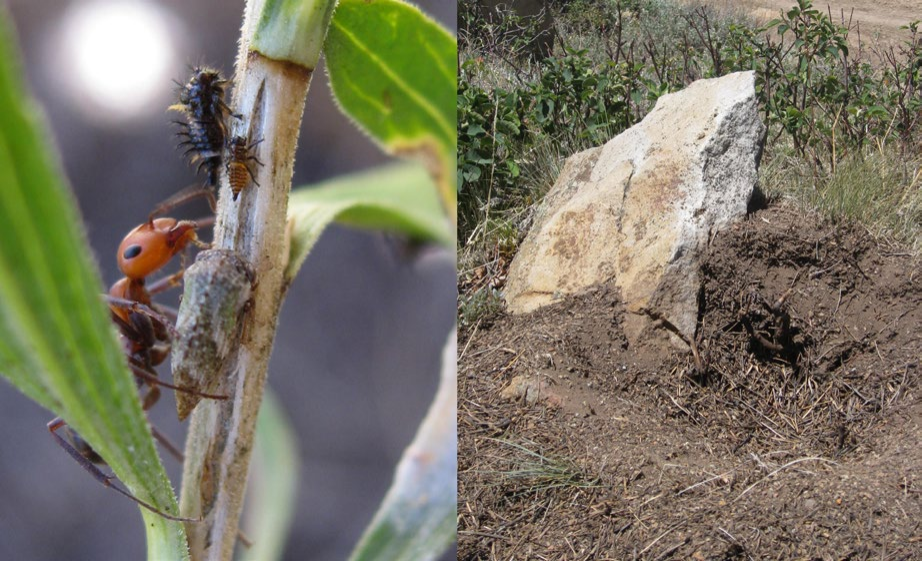
Images of the study organisms in Almont, Colorado. On the left, a thatch ant (*Formica obscuripes*) collects sugary excrement called “honeydew” from treehoppers (*Publilia modesta*) on rabbitbrush (*Chrysothamnus viscidiflorus*). On the right, an ant nest rebuilds following damage by a black bear (*Ursus americanus*). Note the new layer of thatch in the center of the nest; the rock provides a reference for the height of the nest prior to bear damage. Some ant nests become inactive after bear attacks. Photo credit: J. B. Grinath

**Figure 2:**
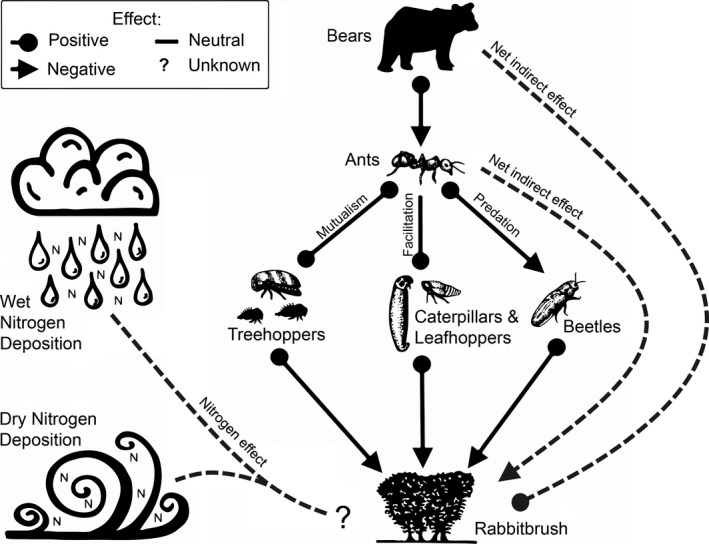
Interaction web for the effect of atmospheric nitrogen (N) deposition on a trophic cascade from bears to plants. Ants engage in a food‐for‐protection mutualism with herbivorous treehoppers and facilitate caterpillars and leafhoppers, which benefit from enemy‐free space provided by ant deterrence of other arthropod predators. Ants are also predators of herbivorous beetles, but ants have a negative net effect on plants because of strong protection for herbivores. Bears indirectly benefit plants by consuming ants, which suppresses protection for herbivores and the negative effects of herbivores on plants. Reciprocal interactions are shown as paths that end in circles for species that benefit from the interaction and arrows for those negatively affected. Indirect effects are dashed, gray paths. The “nitrogen effect” represents the effect of N deposition on the cascade. Drawings by J. B. Grinath

In this study, I performed a N enrichment experiment to examine whether a trophic cascade from black bears (*Ursus americanus*) to rabbitbrush plants (*Chrysothamnus viscidiflorus*) could potentially be influenced by low‐level N deposition. Ants are a staple food for bears (Baldwin & Bender, 2009), especially ants in the genus *Formica* (Auger, Ogborn, Prichett, & Black, 2004; Bull, Torgersen, & Wertz, 2001; Grobe, Kaczensky, & Knauer, 2003; Noyce, Kannowski, & Riggs, 1997; Swenson, Jansson, Riig, & Sandegren, 1999). Across western North America, the thatch ant *Formica obscuripes* (Figure 1) constructs large, mounded nests to house their colonies (Jergensen, Storer, & Risch, 2005; Weber, 1935). A prior study (Grinath, Inouye, & Underwood, 2015) showed that black bears in Colorado tear apart *F. obscuripes* nests to consume the immature and adult ants within, leading to nest inactivity and a cascade of effects on surrounding plants (Figure 2). The ants have a mutualistic (positive) relationship with a dominant herbivore, a sap‐sucking treehopper (*Publilia modesta*: Figure 1) which provides sugary honeydew as food in exchange for ant protection from other arthropod predators, such as lady beetles and spiders (Grinath et al., 2015). Ant protection for treehoppers reduces predator abundances, which facilitates other herbivores that are unmolested by ants, such as caterpillars and leafhoppers (Grinath et al., 2015). The ants are also predators of leaf‐chewing beetles (*Monoxia schyzonycha*), but the ants’ net effect on plants stems mostly from mutualistic and facilitative interactions with herbivores, resulting in reduced plant performance (Grinath, Inouye, Underwood, & Billick, 2012). Bears decrease ant protection for herbivores, which allows other arthropod predators to suppress herbivores and benefit plant reproduction and growth (Grinath et al., 2015).

Previous work with the same ant and treehopper species, but a different host plant, found that high‐level N enrichment intensified indirect interactions between ants and plants (Strauss, 1987). Within a single growing season, fertilization of sagebrush (*Artemisia ludoviciana*) with N resulted in increased abundances of honeydew‐producing herbivores and honeydew‐tending ants, which drove a large reduction in beetle abundances and chewing damage to plants (Strauss, 1987). Though the net effect of ants on sagebrush plants is unclear from this prior study, I expected that low‐level N enrichment would enhance herbivore survival and thus cause stronger cascades of effects from ants to rabbitbrush that would be detrimental for plant performance. As a consequence of these dynamics, I hypothesized that bears would be more beneficial for rabbitbrush under N‐enriched conditions. Alternatively, N fertilization could result in a weaker cascade if plants become more resistant to herbivores by enhancing plant defenses or tolerance to herbivory (Schmitz, 1994; Schmitz, Hamback, & Beckerman, 2000). Experiments manipulating multiple soil nutrients have demonstrated that cascades from ants to plants can become weak as plant resistance to herbivory increases with fertilization (Mooney, Halitschke, Kessler, & Agrawal, 2010), however, it is unclear whether this alternative hypothesis is supported for N enrichment (independent of other nutrients) and trophic cascades in systems with mutualist ants and herbivores.

I used a field experiment to test for relationships between low‐level N fertilization and bear effects on plants and to identify changes in the species interactions comprising the trophic cascade (Figure 2). For three summers, I added N (5 kg ha^−1^ year^−1^) to ant nest enclosures that delineated foraging areas for ants and that experienced very low ambient N deposition (~2 kg ha^−1^ year^−1^). I also documented whether bears caused ant nest inactivity within the enclosures, which I used to represent bear effects on the plant–arthropod community. In the third summer, I manipulated the presence of treehoppers and foraging ants on rabbitbrush plants in the enclosures, and analyzed rabbitbrush performance (reproduction and growth) and insect abundance responses to N additions, bear‐induced nest inactivity, foraging ants, and treehoppers. Altogether, this study provides an initial test for the effects of short‐term, low‐level N deposition on trophic cascades.

## 2 MATERIALS AND METHODS

### 2.1 Experimental setup

I conducted this investigation in a montane meadow near Almont, Colorado. The study system is described in Figures 1 and 2; further details are provided in previous experiments in this system (Grinath et al., 2012, 2015). To evaluate the effects of N enrichment from plants to ants, I installed 36 ant enclosures in the meadow during the summer of 2010 to mimic conditions of N deposition. These enclosures were constructed at the scale of meadow patches that support a single ant nest and established a foraging arena for each nest. Made from smooth plastic landscape edging (15 cm tall) buried 5 cm deep, each enclosure was 5 m in diameter and encircled a central *F. obscuripes* nest. Colonies of this ant species are polydomous, occupying from one to hundreds of nests (Herbers, 1980; McIver, Torgersen, & Cimon, 1997; O'Neill, 1988), and in this area of Colorado, separate *F. obscuripes* nests can be as close as 2.36 m apart (Conway, 1996). Thatch ants primarily forage near their nests (Herbers, 1980; McIver & Yandell, 1998); thus, a foraging radius of 2.5 m around a nest was considered sufficient for sustaining a single *F. obscuripes* nest. From 2010 to 2012, these enclosures were maintained each summer by reinstalling barriers and removing bridging vegetation. The plastic barriers were slippery for thatch ants, which could not crawl over the material, but foraging trails were occasionally dug underneath the barriers. Enclosures were inspected weekly throughout the summer and all such foraging trails were immediately eliminated by filling in tunnels and wiping away chemical markers. By mid‐summer, the enclosures were effective in containing thatch ants, but smaller ant species and other arthropods were able to climb over the barriers, as well as jump, float or fly in and out of the enclosures.

From 2010 to 2012, I simulated elevated N deposition (3.5 times ambient) in half of the ant enclosures. Total N deposition in the meadow was approximately 2 kg N ha^−1^ year^−1^ when the study began in 2010, based on the rate at nearby Gothic, CO (Clean Air Status and Trends Network, US Environmental Protection Agency). To mimic a total N deposition rate of 7 kg N ha^−1^ year^−1^, which is common in the western US (Fenn et al., 2003; Greaver et al., 2012), I applied 0.5 g N m^−2^ of slow‐release ammonium nitrate fertilizer once each summer to randomly‐selected enclosures (*n* = 18). Though N deposition can have non‐fertilization effects, such as being directly toxic to plants, montane ecosystems are typically limited by N availability and changes in plants are driven primarily by soil fertilization (Bassin, Volk, & Fuhrer, 2013; Bobbink et al., 2010; Helliwell, Britton, Gibbs, Fisher, & Aherne, 2008). Similarly, I expected effects N enrichment on insects to be mediated by N effects on plants (Throop & Lerdau, 2004). Therefore, the experimental simulation was able to produce conditions similar to elevated rates of N deposition. Nitrogen was applied using a stratified experimental design, with treatments randomly assigned within groups of 12 nest enclosures in three sections of the study meadow (east, central, and west).

Bears foraged on ant nests within the enclosures from 2010 to 2012, and I determined nest inactivity in September, 2012 as an index of bear effects on the plant arthropod community. As in a prior study (Grinath et al., 2015), nests were considered inactive if no more than one ant‐worker emerged when the ground next to the nest was beaten. Ant nest inactivity occurs when nests are severely damaged by bears and are either immediately destroyed or exposed to environmental conditions or pathogens that subsequently cause inactivity; some nests recovered from minor bear damage and remained active. Of the 36 focal ant nests, bears caused nest inactivity in four enclosures with ambient N and three enclosures with N additions. Bear attacks on nests and nest inactivity occurred in all three sections of the study meadow and were unrelated to N additions according to binomial models (incidence of bear attacks: χ^2^ = 0.178, *p* = 0.673; nest inactivity: χ^2^ = 0.224, *p* = 0.629). Too few ant nests were unmolested by bears during the experiment (*n* = 5) to assess the effects of “bear attacks” as an independent variable, but replication was adequate to evaluate bear‐induced nest inactivity as a factor that was effectively crossed with the N manipulation. Previous study in this system indicates that foraging ants from neighboring nests do not compensate for the loss of ants due to bear predation (Grinath et al., 2015); therefore, nest inactivity within the ant enclosures represented natural conditions.

To test for N enrichment effects on each link in the cascade between bears and plants, I factorially crossed manipulations of foraging ant and treehopper presence on individual rabbitbrush in 2012. Four rabbitbrush in each enclosure (total = 144 plants) with similar flowering bud phenology and size were chosen for experimentation. Ant and treehopper treatments were randomly assigned within each enclosure. Foraging ants were excluded from plants with sticky “tanglefoot” barriers (Scotts Company, Marysville, OH) at the base of plants. Treehopper abundances were adjusted to obtain an initial presence of 60 nymphs per plant on July 17; treehopper absence was maintained manually at the start of the experiment and in three surveys over the following three weeks. Treehopper nymphs are sedentary and abundances were adjusted after most nymphs had been born, but a few additional nymphs may have been born on plants or migrated from senescent host plants during the experiment (Reithel & Campbell, 2008). Vegetation surrounding each plant was trimmed to limit ant access and treehopper migration.

### 2.2 Data collection

I collected data on plant performance traits representing reproduction (seed production per initial flower bud) and growth (change in aboveground mass). Initial flowering bud abundance, representing potential reproduction, was measured on July 19, 2012. Rabbitbrush seed production was measured by bagging flowers in mesh at the end of the arthropod surveys and collecting the mature seed on October 2. The seed was sorted from other plant materials and weighed. Reproduction was then measured as mg seed per initial flower bud. Aboveground plant growth was obtained by calculating the difference between plant mass estimates on July 11 and August 3. These estimates were found nondestructively by applying measures of plant height and cover area to an allometric model developed previously for rabbitbrush at this site (Grinath et al., 2015). Cumulative abundances were measured to evaluate insect herbivore (treehoppers, beetles, caterpillars, and leafhoppers) and foraging ant (which persisted in exclosures after ant nests became inactive) responses across the duration of the experiment. These measures were obtained by surveying the individual experimental plants for insects three times from July 19 to August 3 and summing the abundances across surveys. The aboveground portion of two plants was removed by a large herbivore during the experiment; thus, data were collected for a total of 142 plants.

### 2.3 Data analysis

Plant and insect responses were analyzed with generalized linear mixed effects models (GLMMs) (Zuur, Ieno, Walker, Saveliev, & Smith, 2009) in R v3.4.4 (R Core Team, 2018). Individual plants were nested within random ant enclosures and the fixed explanatory factors were the presence of treehoppers, foraging ants, bear‐induced nest inactivity, and N additions (all binary factors). Package “nlme” was used for GLMMs with Gaussian‐distributed residuals because these models can correct for unequal variances across experimental groups; package “lme4” was used for GLMMs with other error distributions (using the “bobyqa” optimizer option to achieve model convergence). For Gaussian‐distributed models, I used a model selection procedure based on Akaike Information Criterion (AIC) scores to identify the best random variable structure and variance structure for each model (Zuur et al., 2009). All models had full fixed variable structures comprising all main effects and interaction terms. In the selection procedure, I first compared models with random intercepts versus random slopes and intercepts; then among models with fifteen variance structures (varIdent specified for all factor combinations and each main effect) to account for unequal variances across experimental groups. Best models were identified as having AIC scores at least two units lower than competing models.

I used Gaussian‐distributed models for plant responses to the experimental factors. I also used Gaussian GLMMs for treehopper and foraging ant abundance responses to N, nest inactivity and mutualist partners (ants or treehoppers, respectively) because these data were approximately normally distributed and the models could account for unequal variances. Qualitatively equivalent results were found for treehoppers and ants in Poisson and Negative Binomial GLMMs, indicating that the Gaussian GLMMs were robust to deviations from normality in the data. For other count data (beetles, leafhoppers), I compared models with Poisson versus Negative Binomial distributions; if too few individuals were observed to conduct these models (caterpillars), data were converted to presence/absence and analyzed with a Binomial GLMM. The residuals of final models were visually assessed to confirm that they met model assumptions. In two cases (plant reproduction and growth), extreme outlying observations were identified in boxplots as points greater than three times the inter‐quartile range above the third quartile of the entire dataset; likely due to measurement error, these points were deleted to meet model assumptions. In all models, significance was determined by analyses of deviance (package “car”) with type II SS to account for the unbalanced experimental design. Explanatory factors were considered statistically significant if *p* ≤ 0.05 and marginally significant if *p* ≤ 0.10.

I evaluated Tukey post hoc contrasts to understand differences between experimental groups indicated by significant GLMM results. These contrasts were conducted with the “multcomp” package with the fixed structure of the best model identified above modified to focus on the interaction of concern. After finding a significant nest inactivity × N enrichment interactive effect for plant reproduction, I also conducted a post hoc analysis to evaluate whether this result was influenced by low replication for plots with inactive ant nests. I reran the GLMM to re‐assess the nest inactivity × N result when individual plots within the experimental group for inactive ant nests and ambient N conditions were removed from consideration (four models total, one for each plot removed). A significant interaction between the nest inactivity and N factors in these models would indicate that the results were robust and not dependent on data from a single plot within the experimental group. Furthermore, these results would indicate whether three replicate plots with inactive ant nests were sufficient for detecting nest inactivity effects on plants within the N treatments.

To further understand how N enrichment affected interactions across the ecological network, I used a multi‐group structural equation model (SEM) (Grace, 2006) to analyze differences in cascades from ants to plants in ambient and enriched N conditions. Similar to previous work (Grinath et al., 2012, 2015), I analyzed per capita effects of ants on herbivores (treehoppers, leafhoppers and beetles) and of herbivores on plant seed production (Supporting information Figure S1). Initial flower bud abundance was included as a covariate representing potential seed production and plant quality early in the growing season. I included paths from flower buds to seed production and to herbivores to further evaluate changes in plant–herbivore relationships. As in previous SEM analyses (Grinath et al., 2012, 2015), “change in treehoppers” (treehopper abundances in the final survey minus those at the start of the experiment) was used as a response variable representing treehopper survival because initial abundances were manipulated. Prior to analysis, I visually examined bivariate relationships among the SEM variables to determine if there were extreme outlying observations. One plant‐arthropod community with extreme data values was removed to meet model assumptions; subsequent evaluation confirmed that the removal of this observation did not change the qualitative interpretation of the SEM analysis.

In addition to the focal paths, I considered including paths between herbivores (Supporting information Figure S1a) and used a model pruning strategy to determine whether to include these paths. Paths between herbivores were modeled as non‐directional covariances/correlations because causal relationships were unclear between herbivores, whereas other paths in the models represented directional, causal hypotheses. Using the full dataset, each of the non‐focal paths was deleted and model fit was compared to that of the full model (described in Grinath et al., 2015). This procedure indicated that non‐focal paths between herbivores did not contribute to model fit (Supporting information Table S1); thus, the best‐fit model structure did not include paths between herbivores (Supporting information Figure S1b). This model structure was then applied to data for plant–insect communities in ambient and enriched N conditions. Replication was adequate to compare these two groups, but there were too few observations to conduct models separately for each nest inactivity × N experimental group (Grace, Scheiner, & Schoolmaster, 2015); the effect of bear‐induced nest inactivity in ambient and enriched N conditions can be interpreted from changes in ant abundance in these models. Final model fit was assessed with χ^2^ lack‐of‐fit tests and raw and standardized per capita and net effects were calculated for each condition. Because paths represented directional hypotheses for positive and negative effects (Figure 1), we considered paths to be significant if *p* ≤ 0.10, which is analogous to interpreting one‐tailed tests and appropriate when complimentary analyses (GLMMs) provide support (Grace, 2006; Grinath et al., 2012, 2015). The multi‐group SEM was conducted in Amos 5.0.1 (Arbuckle, 2003).

## 3 RESULTS

**Figure 3:**
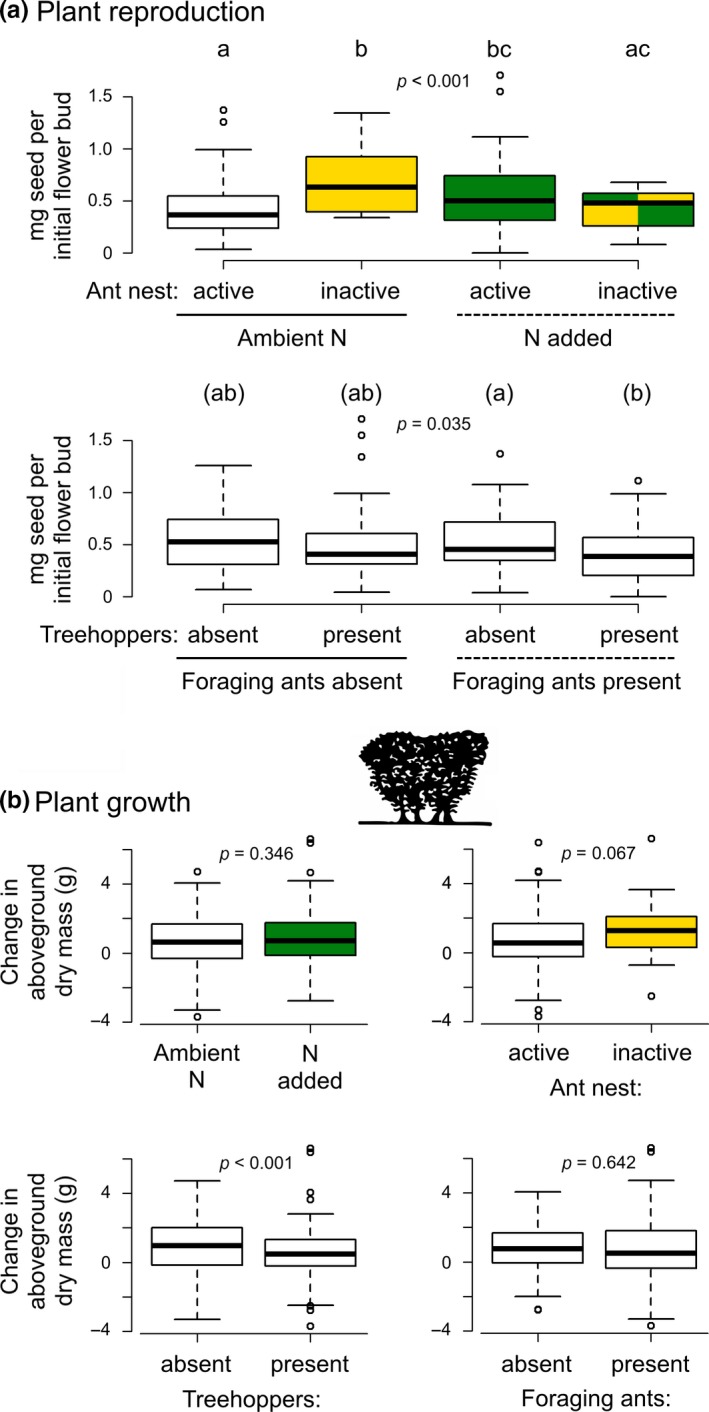
Rabbitbrush (a) reproduction and (b) growth responses to N, treehopper and foraging ant manipulations, and bear‐induced ant nest inactivity. Data are shown as boxplots and *p*‐values are for GLMM results. Lowercase letters denote post hoc contrasts in (a). Contrasts without parentheses are significant at *p* ≤ 0.05, while the contrast shown with parentheses has *p* ≤ 0.101

Low‐level N enrichment and bear‐induced ant nest inactivity had interacting effects on plant reproduction (Figure 3a; Supporting information Table S2). In ambient N conditions, nest inactivity resulted in increased plant reproduction (seed production per initial flower bud), but plant reproduction was unchanged across enclosures with active and inactive ant nests in N‐enriched conditions. Post hoc evaluation indicated that this result was robust, with adequate power to detect a similar or stronger effect of nest inactivity on plants under enriched N conditions if it were present (Supporting information Table S3). In addition, there were interacting effects of treehopper and foraging ant manipulations on plant reproduction, though post hoc evaluation provided weak support (Figure 3a). Plant reproduction tended to decrease in the presence of both treehoppers and ants, compared to when only ants were present. For plant growth, N fertilization had no effect, but nest inactivity tended to have a positive effect (χ^2^ = 3.360, *p* = 0.067) while treehoppers had a negative effect (Figure 3b; Supporting information Table S2). Foraging ant manipulations did not influence plant growth.

**Figure 4:**
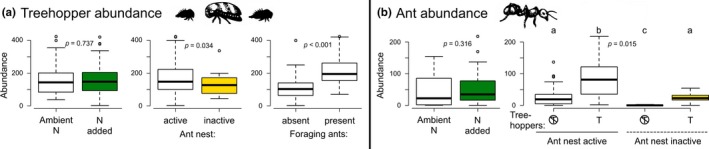
Responses of mutualist (a) treehoppers and (b) ants to N enrichment, bear‐induced ant nest inactivity, and corresponding mutualist partners. Data are depicted as boxplots and *p*‐values are for GLMM results. In (b), treehopper presence treatments are designated by “T” underneath boxplots, with crosses indicating treehopper absence. Lowercase letters denote significant (*p* ≤ 0.05) post hoc contrasts in (b)

Abundances of treehoppers, the numerically dominant herbivore, were unaffected by N additions (Figure 4a; Supporting information Table S4), indicating that survival was unchanged. Treehopper abundances were 89% greater in the presence of foraging ants and were 22% lower when ant nests were inactive (Figure 4a). Foraging ant abundances were also unaffected by N fertilization, but responded to interactive effects of bear‐induced nest inactivity and treehoppers (Figure 4b). Across the experimental treehopper and nest inactivity conditions, ants attained highest abundances on plants when mutualist treehoppers were present and nests were active. Foraging ant abundances were similar when nests were active and treehoppers were absent and when nests were inactive and treehoppers were present. Lowest foraging ant abundances occurred when nests were inactive and treehoppers were absent.

**Figure 5:**
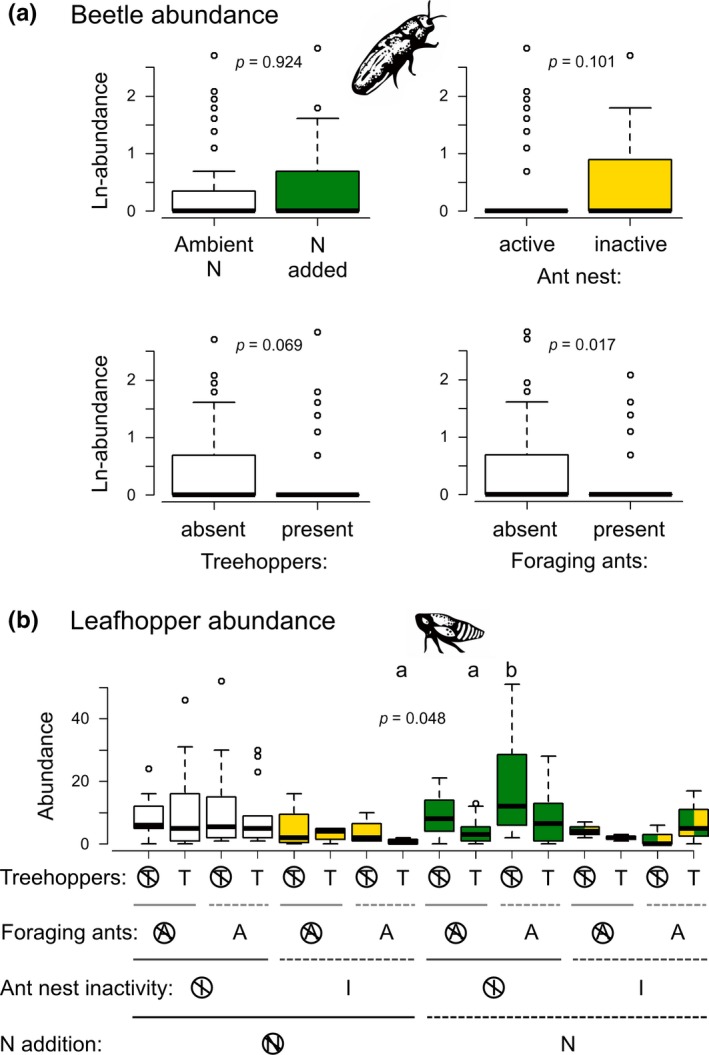
Responses of herbivorous (a) beetles and (b) leafhoppers to N, treehopper and foraging ant manipulations, and bear‐induced ant nest inactivity. Data are depicted as boxplots; crosses on symbols underneath boxplots in (b) signify the absence of the corresponding experimental condition. *p*‐values are for GLMM results. Lowercase letters denote significant (*p* ≤ 0.05) post hoc contrasts in (b); all other comparisons were not significant and are not indicated in the figure

Other herbivore groups had variable responses to N enrichment and the other experimental factors (Figure 5; Supporting information Table S5). Beetle abundances decreased in the presence of foraging ants, but were unaffected by N additions (Figure 5a). Beetle abundances tended to be lower in the presence of treehoppers as well (χ^2^ = 3.305, *p* = 0.069), and there was a trend toward greater numbers of beetles when ant nests were inactive (χ^2^ = 2.686, *p* = 0.101). Caterpillar presence on plants was not affected by the experimental factors (Supporting information Table S5), but leafhopper abundances responded to a four‐way interaction involving all experimental factors (Figure 5b). The post hoc evaluation of this interaction was complicated, but the clearest result was that leafhoppers achieved highest abundances when N was added, foraging ants were present (in enclosures with active nests) and treehoppers were absent.

**Figure 6:**
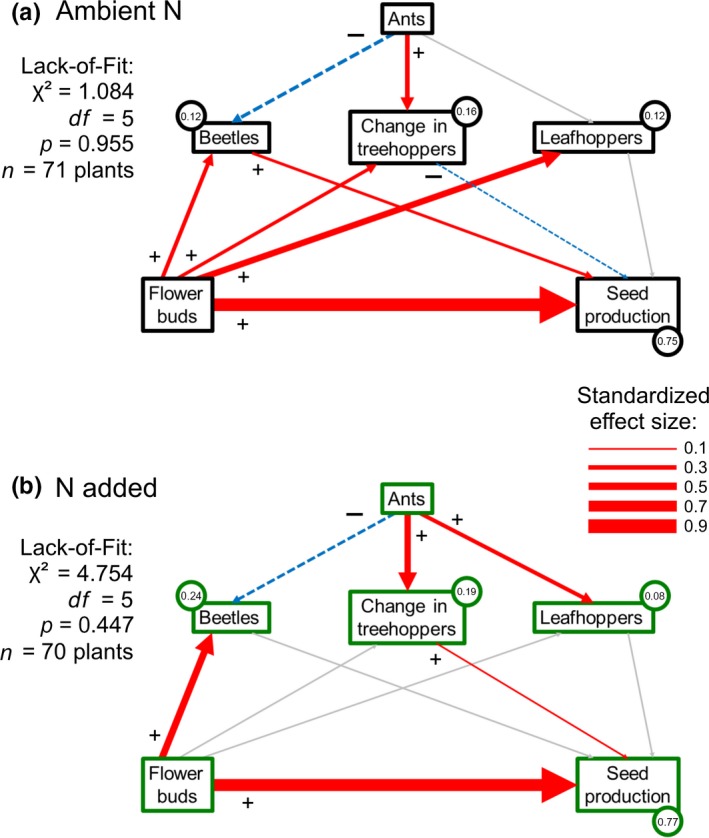
Multi‐group SEM results comparing interaction strengths under (a) ambient and (b) enriched N conditions. Red and blue paths are significant (*p* ≤ 0.10) per capita effects, with line thickness illustrating the standardized effect size. Positive effects are red, solid arrows, and negative effects are blue, dashed arrows, with the sign of the interaction indicated at the base of each path. Thin, gray arrows are nonsignificant paths. Endogenous (dependent) variables are boxes with *R* ^2^ values provided in adjoining circles; exogenous (independent) variables are boxes lacking circles

To understand changes in the cascade between bears and plant reproduction (Figure 3a), I used multi‐group SEM to analyze component cascades from ants to plants in the absence and presence of N enrichment. Diagrams of the SEMs with standardized per capita effects are shown in Figure 6, summaries of raw and standardized per capita and net effects and bivariate relationships are provided in the supporting information (Tables S6, S7; Figure S2). Models for both N conditions passed lack‐of‐fit tests (Figure 6), indicating that the model structure fit the data adequately. In ambient N conditions (Figure 6a), ants had a negative net effect on plant seed production (Supporting information Table S7) that was due to the positive effect of ants on treehoppers and the negative effect of treehoppers on seeds. Ants also suppressed beetles, which may have indirectly benefited plants, but beetles had an unexpected positive relationship with seed production. Ant effects on leafhoppers and leafhopper effects on seed production were nonsignificant. In addition, flower bud abundance had positive effects on all herbivores under ambient N.

The interaction strengths in the ecological network were very different when N was added. In fertilized conditions (Figure 6b), ants no longer had a negative net effect on plants (Supporting information Table S7), despite stronger positive ant effects on treehoppers and leafhoppers and weaker ant predation on beetles. Ants did not negatively affect plants because treehoppers had an unanticipated positive relationship with seed production and the leafhopper effect on plant reproduction was nonsignificant. The effects of flower buds on herbivores were also drastically different under N enrichment compared to ambient conditions. Though the positive effect of flower buds on beetle abundances intensified, other paths from flower buds to herbivores were nonsignificant. In sum, the SEM analysis indicates that the cascade was dampened in N‐enriched conditions because plant–herbivore interactions became weak, thereby weakening the indirect net effect of ants on plants.

## 4 DISCUSSION

This study shows that even short‐term, low‐level N deposition can alter the relative strength of resource and consumer forces in ecological communities, which has consequences for plant performance. Ecologists have long debated the relative importance of resources and consumers for structuring species abundances and biomass in communities (Cebrian, 1999; Estes et al., 2011; Gruner et al., 2008; Hairston et al., 1960; Hall, Shurin, Diehl, & Nisbet, 2007; Power, 1992; Ripple et al., 2014; Schmitz, 1994; Schmitz et al., 2000; Strauss, 1987). The present study demonstrates that N deposition mainly affects interactions between lower trophic levels and is consistent with a growing consensus that fertilization primarily benefits plants and not consumers (Borer, Halpern, & Seabloom, 2006). Nevertheless, it can be difficult to predict how N deposition will influence consumer control of community structure because it is often unclear how N enrichment will affect plant resources for herbivores and indirectly impact higher trophic levels.

Contrary to my expectation, low‐level N enrichment caused the trophic cascade between bears and plants to become weak. This result is consistent with other fertilization studies that manipulated multiple nutrients (including N), which have shown that cascades often do not become stronger with fertilization (Borer et al., 2005). Though previous study of ants and treehoppers on sagebrush found that interactions between ants, herbivores, and plants intensified in N‐enriched conditions (Strauss, 1987), that study did not evaluate the net effect of ants on plant performance and it was unclear whether greater abundances of honeydew‐producing insects resulted from greater reproduction or survival. Here, I found that the beneficial effects of ants on herbivores became stronger with N fertilization, even though treehopper and tending ant abundances did not significantly increase. This result suggests that the protective benefits of ants for herbivores were greater with N enrichment, potentially due to increased predation pressure from other arthropod predators (Cushman & Whitham, 1989; Grinath et al., 2015). Unlike a recent study in a similar system assessing high‐level N enrichment (Pringle, Ableson, Kerber, Vannette, & Tao, 2017), the present study found that indirect effects of ants on plants were not independent of N additions.

While ants had stronger positive effects on herbivores, most interactions between herbivores and plants became weak in N‐enriched conditions. In ambient N conditions, treehoppers negatively affected plant reproduction, providing the final link in the cascade of effects on plants. When bears suppressed ant abundances (Figure 4b), ant‐treehopper and treehopper–plant interactions were also suppressed (Figure 6a), ultimately benefitting plant reproduction (Figure 3a). However, when N was added to the system, the SEM analysis indicates that there was a positive relationship between treehoppers and plant reproduction. This positive interaction could suggest that rabbitbrush overcompensated for damage due to treehoppers. More likely, the positive relationship could result from treehoppers benefitting from plants that have greater seed production or some unmeasured aspect of plant vigor. When significant results are found in SEMs that are the opposite sign of the hypothesis, causality is unclear and requires further study to resolve (Grinath et al., 2015). Regardless of the mechanism responsible for this positive relationship, it is clear that treehoppers were no longer detrimental for plant reproduction under N fertilization, which caused the cascade from bears to plants to attenuate.

This study is consistent with the hypothesis that trophic cascades become weak as plant resistance to herbivory increases with N enrichment (Schmitz, 1994; Schmitz et al., 2000). Plants can increase their resistance to herbivory by investing resources in anti‐herbivore defenses and/or tolerance mechanisms that compensate for herbivore damage. Rabbitbrush contain C‐based metabolites, such as terpenes, coumarin glucosides and flavonoids (Ahmed et al., 2006), that may provide defense against herbivores (Gershenzon & Dudareva, 2007; Throop & Lerdau, 2004) and could be promoted by greater photosynthetic capacity in N‐enriched environments (Nunes‐Nesi, Fernie, & Stitt, 2010). Alternatively, N enrichment could allow rabbitbrush to replace resources lost to herbivores, making the plants more tolerant to herbivory. Additional study is necessary to understand the mechanisms driving N‐induced changes in plant–herbivore interactions, especially in relation to plant resistance to herbivores.

The results of this study support previous research showing that, by consuming ants, black bears indirectly affect plant performance (Grinath et al., 2015). Prior work in the same study system found that the cascade of effects from bears to plants was mediated by the positive effect of ants on leafhoppers and a negative effect of leafhoppers on plants, but that in other years, ant effects on plants occurred through treehoppers (Grinath et al., 2012, 2015). The present study clarifies that bears can indirectly affect plants through a cascade involving the ant‐treehopper mutualism and the negative effect of treehoppers on plants. Ants still benefited leafhoppers in the current study, but this effect did not indirectly influence plants. Though there is variation between years in the herbivore species that are most damaging for plants, the net effects of ants and bears on plants appear qualitatively stable through time as ant benefits to herbivores consistently outweigh ant predatory effects on herbivores. Across western North America, black bears are sympatric with the ant, herbivore, and plant species that compose this cascade (Cushman & Whitham, 1989; Jergensen et al., 2005; Tilley & St. John, 2012), and there is great potential that bear consumption of ants indirectly affects the performance of rabbitbrush and other plant species in many areas. However, the present study also indicates that we should expect the strength of these trophic cascades to vary across space and time due to changing nutrient availability for plants.

Elevated rates of N deposition are now common across the globe (Dentener et al., 2006; Duce et al., 2008; Vitousek et al., 1997) and have already reshaped many ecosystems (Bobbink et al., 2010; Clark, Morefield, Gilliam, & Pardo, 2013; Maskell, Smart, Bullock, Thompson, & Stevens, 2010). Changes in plant communities due to N deposition are often attributed to altered competitive dynamics that favor nitrophilous plants (Hautier, Niklaus, & Hector, 2009; Stevens, Dise, Gowing, & Mountford, 2006; Suding et al., 2005). The present study suggests that some of these changes are due to altered dynamics between plants, herbivores, and higher trophic levels. This study shows that a cascade can change after only three years of low‐level N deposition. The long‐term consequences of N deposition are likely to have more dramatic effects (Clark & Tilman, 2008). For example, long‐term exposure to N enrichment results in greater insect herbivore abundances and damage to heather plants (Kerslake, Woodin, & Hartley, 1998; Taboada, Marcos, & Calvo, 2016), which contributes to the conversion of heathlands to grasslands (Terry, Ashmore, Power, Allchin, & Heil, 2004). Though short‐term N fertilization may increase rabbitbrush resistance to herbivores, continued N enrichment could result in greater herbivore and ant abundances and reduced rabbitbrush performance, with negative effects on rabbitbrush dominance within the ecosystem. Conservation of bears and other omnivores and predators that generate trophic cascades may be essential for managing the long‐term repercussions of N deposition, which deserves further study. Management of trophic cascades could be key to stabilizing ecological communities and the ecosystem functions and services that are threatened by anthropogenic nutrient enrichment (Compton et al., 2011; Hautier et al., 2014; Isbell et al., 2013).

## CONFLICT OF INTEREST

None declared.

## AUTHOR CONTRIBUTION

JBG performed all parts of this study.

## DATA ACCESSIBILITY

Data from this study are available from the Dryad Digital Repository: https://doi.org/10.5061/dryad.30nj0tb (Grinath, 2018).
