# Supplementary material for: Short‐term, low‐level nitrogen deposition dampens a trophic cascade between bears and plants
Source: Ecol Evol. 2018 Oct 25;8(22):11213–23. doi: 10.1002/ece3.4593 (PMC6262928; doi:10.1002/ece3.4593)
Supplement: Supplementary file 1 [file ECE3-8-11213-s001.docx]

**Supporting Information**

Title: Short-term, low-level nitrogen deposition dampens a trophic cascade between bears and plants

Author: Grinath, J. B.

**Contents:**

Table S1. Model pruning strategy for the multi-group SEM

Table S2. Generalized linear mixed effects model results for plants

Table S3. Post hoc GLMM results for plant seed production

Table S4. GLMM results for treehoppers and ants

Table S5. GLMM results for herbivorous beetles, caterpillars and leafhoppers

Table S6. Per-capita effects for the multi-group SEM

Table S7. Net effects for the multi-group SEM

Figure S1. Structure of the SEMs before and after model selection

Figure S2. Scatterplots for variables in the multi-group SEM

______________________________________________________________________________

Table S1. Model pruning strategy for the multi-group SEM analysis.

Table notes: We perturbed non-essential paths to find the best model. The criterion was that paths would be retained if their deletion caused significant χ²-values (*P* ≤ 0.10) or caused Akaike’s information criteria (AIC), root mean error of approximation (RMSEA), and expected cross-validation index (ECVI) values to increase over the full model (at least 2/3 of indices). All models had *n* = 141.

Table S2. Generalized linear mixed effects model (GLMM) results for plant growth and reproduction responses to treehopper, foraging ant, and nitrogen manipulations, and the incidence of bear-induced ant nest inactivity.

Table notes:

One degree of freedom was used for each explanatory factor term in all models. **Bold** statistics have *P* ≤ 0.10.

In columns for variance structure and additional effects: T = treehoppers, A = ants, I = nest inactivity, and N = nitrogen factors.

Model variance structure was computed using the ‘varIdent’ function in the R package ‘nlme’ (v3.1-137).

Table S3. Post hoc generalized linear mixed effects model (GLMM) results for plant reproduction (mg seed per initial bud) responses to nitrogen manipulations, bear-induced ant nest inactivity, and their interactive effects. Plots removed had inactive ant nests due to bear damage and were in ambient nitrogen conditions. Significant statistical interactions (P ≤ 0.05) support the conclusion that the result of the full model (including all plots) is robust and experimental power was sufficient.

Table notes:

One degree of freedom was used for each explanatory factor term in all models. **Bold** statistics have *P* ≤ 0.05.

All models used a variance structure allowing variation among all nest inactivity x nitrogen experimental groups (‘varIdent’ function in the R package ‘nlme’ v3.1-137); residuals were visually assessed to conform to approximately Gaussian distributions.

Plants (*n* = 127) were nested within ant nest (*n* = 35), which was a random term.

Table S4. Generalized linear mixed effects model (GLMM) results for treehopper and foraging ant responses to mutualist and nitrogen manipulations, and the incidence of bear-induced ant nest inactivity.

Table notes:

One degree of freedom was used for each explanatory factor term in all models. **Bold** statistics have *P* ≤ 0.10.

In columns for variance structure and additional effects: T = treehoppers, A = ants, I = nest inactivity, and N = nitrogen factors.

Model variance structure was computed using the ‘varIdent’ function in the R package ‘nlme’ (v3.1-137).

Table S5. Generalized linear mixed effects model (GLMM) results for herbivorous beetle, caterpillar and leafhopper responses to treehopper, foraging ant and nitrogen manipulations, and the incidence of bear-induced ant nest inactivity.

Table notes: One degree of freedom was used for each explanatory factor term in all models. Bold statistics have *P* ≤ 0.10.

Table S6. Raw (covariance) and standardized per-capita effects for the multi-group SEM analysis.

Table notes: **Bold** statistics indicate significant effects (*P* ≤ 0.10).

Per-capita effects are provided as unstandardized (Un.) and standardized (S.) estimates.

Table S7. Raw [and standardized] net effects in the SEMs for plants with (a) ambient nitrogen and (b) nitrogen added.

Table notes: **Bold** statistics indicate that net effects were influenced by significant direct effects (*P* ≤ 0.10).


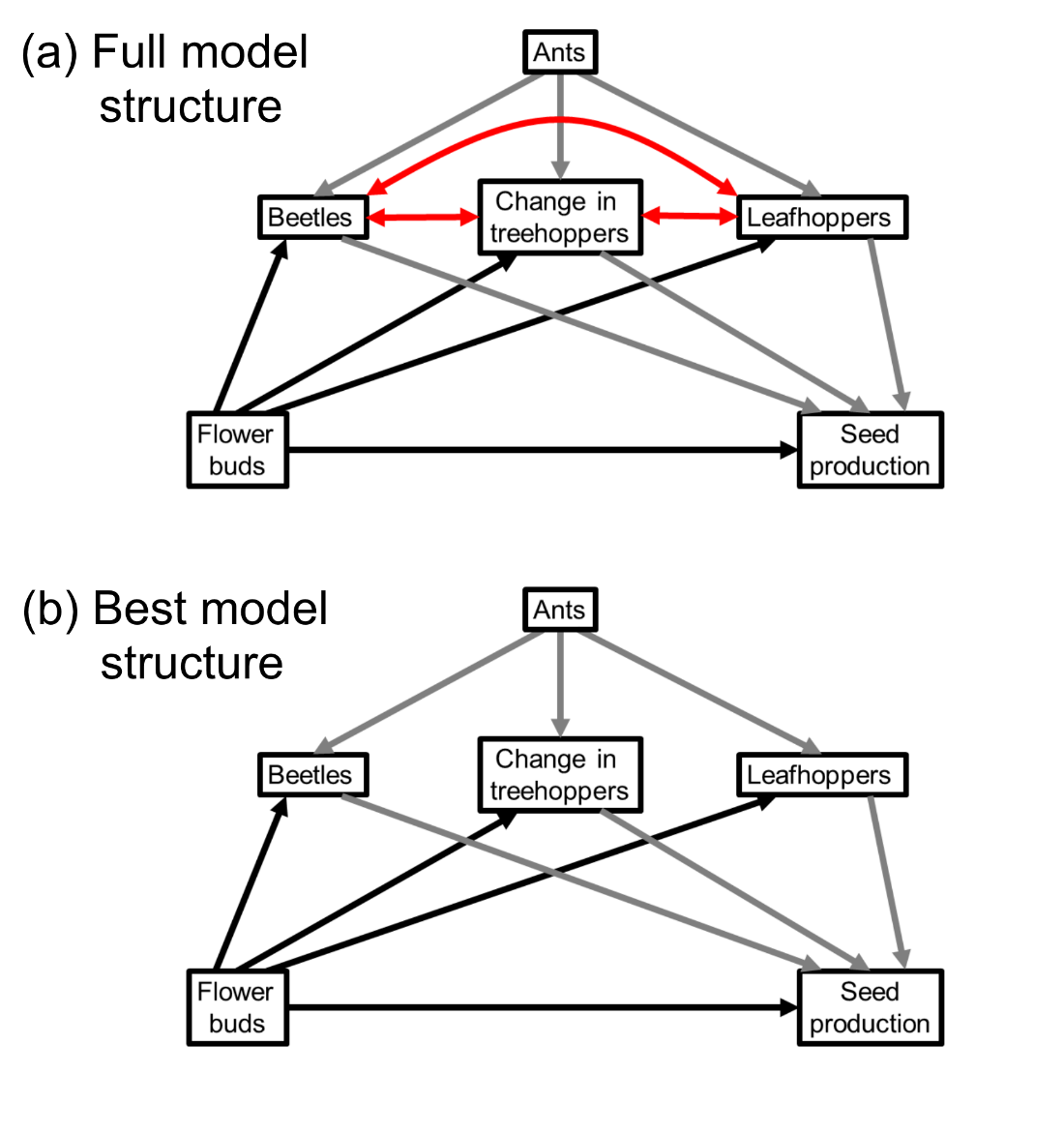


Figure S1. Structure of the SEMs (a) before and (b) after model selection.


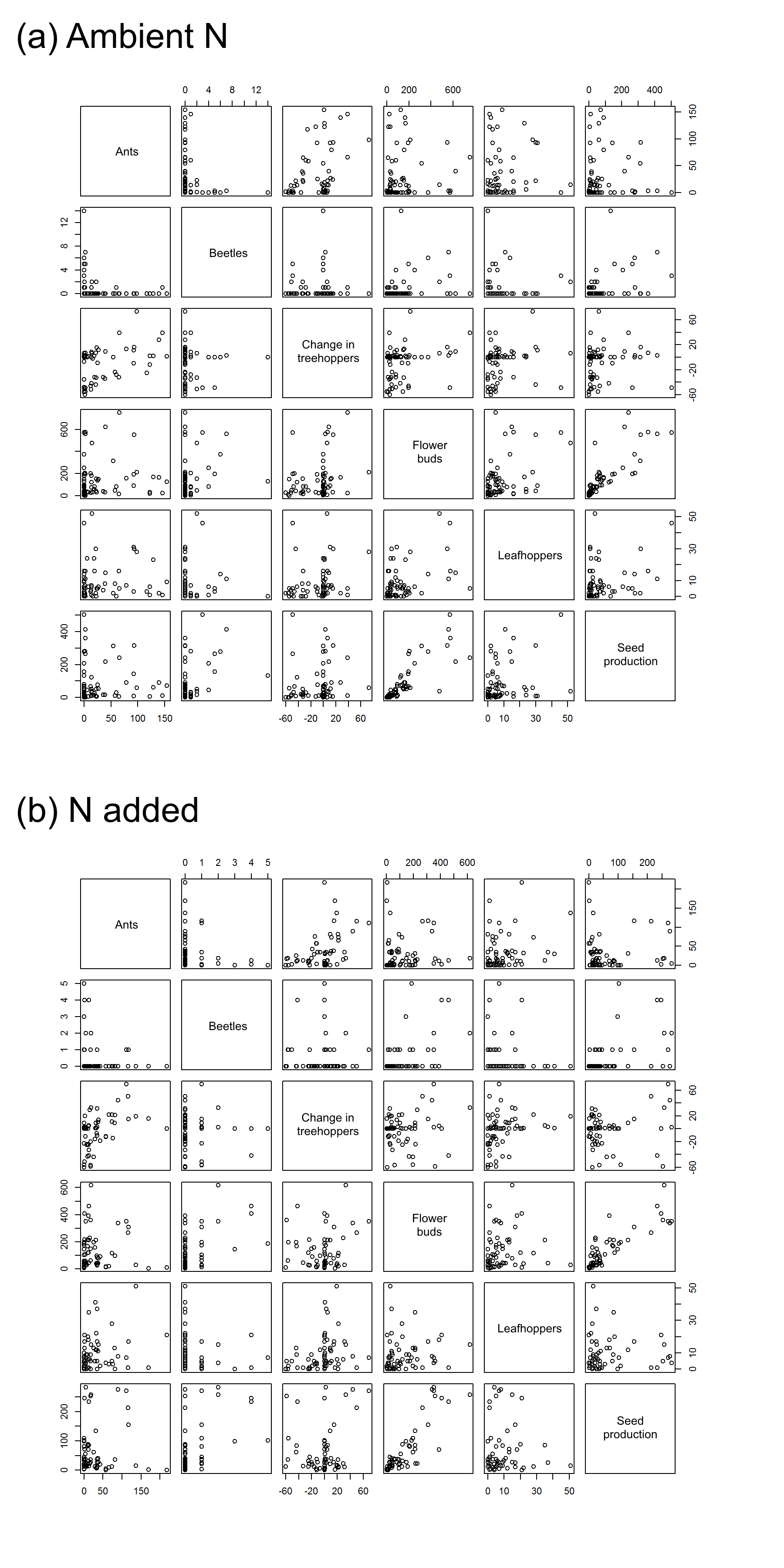


Figure S2. Bivariate scatterplots for variables in the SEMs in (a) ambient nitrogen and (b) nitrogen-enriched conditions.
